# Drugs as Soulmates: The Construction and Validation of a 12-Item Soulmate Scale to Measure Substance Addiction and Loneliness

**DOI:** 10.3390/ijerph17249408

**Published:** 2020-12-15

**Authors:** T. Wing Lo, Jerf W. K. Yeung, Gabriel K. W. Lee, Cherry H. L. Tam, Gloria H. Y. Chan

**Affiliations:** Department of Social and Behavioural Sciences, City University of Hong Kong, Hong Kong; t.wing.lo@cityu.edu.hk (T.W.L.); gabriellkw@gmail.com (G.K.W.L.); ss.hltam@cityu.edu.hk (C.H.L.T.); glo.journal@gmail.com (G.H.Y.C.)

**Keywords:** substance abuse, drug addiction, drug treatment, soulmate, loneliness, Hong Kong

## Abstract

Substance users use substances to tackle psychological stress, frustrations, poor social support and poor-quality relationships. Such experience resembles seeking a soulmate for receiving comfort, a sense of security and satisfaction to relieve feelings of loneliness. Against this backdrop, the study aims to develop a Soulmate Scale to measure substance use and loneliness. Data were collected from 507 drug abusers between 18–71 years of age who were receiving drug addiction treatment in Hong Kong. Both exploratory factor analysis and confirmatory factor analysis were conducted. Results show a valid and reliable scale with three factors: psychological release and shelter, staunch and supportive friendship, and spiritual solace and companionship. This study offers additional support for understanding the drug-taking experience of substance users from their perspective. The Scale provides a useful tool to assess the underlying reasons for substance users to persistently take drugs and formulate corresponding intervention plans to achieve drug abstinence.

## 1. Introduction

Many substance users have experienced consistently poor social support and poor-quality relationships [1]. The need for affiliation easily encourages them to use substances. Substance use is used as a way to secure intimate partnerships and strengthen the identity and membership in a group [2,3]. Even if users are well aware of the potential dangers of substance use, they persist due to the affiliative concerns which are sometimes rather pragmatic and emotional, such as the desire to connect with peers, to increase intimacy, satisfaction and commitment in the relationship and to offset the recognition of risks and harmful consequences [3]. This situation is particularly influential for young women, who perceive intimate relationships as being of high personal significance [4]. Research which investigates the relapse to substance use has found negative mood state and interpersonal conflict as the main reasons for intrapersonal and interpersonal determinants, respectively [5]. Substance use can be a tool for users to cope with traumatic events, such as abuse and victimization [6]. Data from different nations shows that feelings of loneliness, worry, sadness, and hopelessness, as well as a suicide plan are associated with adolescents’ substance use [7].

Recent research identified five stages of drug use [8]. They are: Stage 1: passive user—using drugs for social purposes; Stage 2: active user—expanding the social network; Stage 3: regular abuser—use of drugs as a habit; Stage 4: suspicious abuser—loss of trust in peers; Stage 5: hidden abuser—complete social withdrawal. This process suggests that while affiliative need for a group or an intimate partner would spark substance use in the earlier stages, emotional and social isolation would occur later. Users feel lonely and use substances to handle frustration in human relationships.

Interpersonal and intrapersonal relationships have been identified as having an indirect impact on the consumption of substances through affecting an individual’s emotional intelligence [9]. Moreover, the individual’s poor social ties to others can lead to increased consumption of substances [9]. The use of substances can be a way to establish and maintain relationships with substance-using peers and intimate partners because the refusal of using substances can be regarded as violating their relationships [10,11]. An insecure attachment to the relationships can further predict a high consumption of substances due to a fear of potential rejection and social isolation [11]. Hence, substance users would use substances to sustain a relationship they value.

The motivation for using substances will further be boosted when gratifying benefits are perceived or received [11]. Some psychological perspectives suggested that substance users can obtain a sense of belongingness or “being loved” through the use of substances [12]. The substances can help them relieve pains or fulfil fantasies [12]. Thus, an emotional tie exists between the substances and substance users.

The users will further enter into an unending cycle of hopefulness and despair during the repeated use of substances [9]. The unstable emotions also cause the impulsive consumption of substances [11]. As a result, the relationship between substances and substance users is more than addiction and dependence. It is an undetachable “love yet hate” relationship.

The above literature suggests that substance users use substances to tackle psychological stress, frustrations, and poor social relationships. Such experience resembles seeking a soulmate for receiving comfort, a sense of security and satisfaction to relieve feelings of loneliness. Against this backdrop, the present study aims to develop a Soulmate Scale to measure substance use and loneliness. Particular attention will be given to the affectional, emotional, and relational needs of substance users.

### 1.1. Loneliness

Loneliness has been proven to be associated with unhealthy consequences. It can be a risk factor leading to social isolation and unhealthy behaviors, including smoking and loss of control over gambling [13,14,15,16]. Dependence on substances can be significantly predicted by loneliness [15,16,17]. Furthermore, substance users suffer from more loneliness than the general public [18]. They are trapped in the reciprocal relationship between loneliness and vulnerability to substance use.

The sense of loneliness is associated with various negative psychological factors, such as stress, depression, insecurity, and inferiority [1,19]. The relationship between negative psychological factors and substance use is even reciprocal [16]. Since users adopt substances as a coping strategy to escape from distressing feelings, such as loneliness and social isolation, the successful escape would further reinforce them to continue substance use [1,2,13,20]. In the end, a vicious cycle of substance use persists.

Loneliness is an aversive feeling that desired social interactions are lacking [19]. It is a kind of spiritual and psychic anguish that may lead to addictions [21]. When the perceived loneliness is intensive, unbearable, and even exceeds abject hopelessness, temporary spiritual amelioration and solace produced by substances are perceived as the key to tackling this psychic anguish [6,21], particularly when heavy users have a feeling of disassociation with themselves and the world. Using substances may generate an irreplaceable sense of calm and peace [18,21].

The majority of studies on loneliness and substance use have adopted the UCLA measure which uses a 12-term scale [1]. There are debates on the validity and reliability of the UCLA loneliness scale and other self-constructed scales of loneliness due to the indirect and incomprehensive measures of loneliness, resulting in a challenge of selection and application [1]. The present study does not aim to add anything to the debates. It aims to investigate another issue: When users feel lonely, what do substances mean to them? This inquiry leads to the construction of a new scale, the Soulmate Scale, to understand the situation of substance use caused by loneliness.

### 1.2. Soulmate and Substance Use

When people feel lonely due to psychological distress, they may want to talk to someone who understand them. A soulmate is the perfect candidate. Soulmates need not be romantic partners but can be in a platonic relationship; they can be anyone special (e.g., friends, partners, colleagues) who provides a sense of care, comfort, and safety to the person [22,23]. A soulmate experience normally has the following characteristics: (1) a driving need to be proximal to each other, with separation bringing about pain; (2) receiving a sense of satisfaction, comfort, relief, and security from the other party; (3) having the confidence that the bond will endure regardless of time, distance, or separation; (4) mutual understanding, appreciation, and sharing a common way of seeing things; (5) being able to experience the feelings of the other person even if they are subtle; (6) not being able to deceive or manipulate each other; (7) highly sustainable deep intimacy without emotional barriers; (8) a strangely high level of commonality and synchronicity found in the relationship; and (9) an intuitive feeling that the relationship is significant and of quality [24].

In true friendships, people care about each other because of who they are rather than what they have. They try to collaborate with each other to make the relationship work rather than to focus on the attainment of their own personal benefits [22]. These kinds of relationships can foster deeper intimacy, can survive difficulties and can help cast loneliness away because each party perceives that the other party understands what they think and value [22]. Simply put, a soulmate is a person (1) that one is willing to connect with, care for, and love based on intrinsic motivation rather than extrinsic motivation; (2) from whom one receives unconditional positive regard and understanding of one’s own perspectives, which helps in gaining a sense of comfort, relief, and security that can drive away loneliness and other negative emotions; and (3) who experiences the same spiritual attachment, connection, and commonality with the other party.

Considering the above definitions and criteria of soulmates, substances can play the role of soulmates in the users’ minds. Substances help users to obtain relief and feel comfort and satisfaction when they have a poor relationship with significant others and are coping with a sense of loneliness [13,20]. The substances give them some sort of unconditional positive regard. The spiritual attachment and connection between substances and users is quite similar to falling in love with another person. Research has reported that female users described abstinence from drugs as similar to getting out of the relationship with drugs [4]. Substance use represents a close and emotional attachment between the users and the substances. The former would regard the latter as soulmates who are always available and supportive in times of trouble and willing to listen to ventilation of concerns without any judgement or blame.

## 2. Method

### 2.1. Samples

The study was approved by the Institutional Review Board of City University of Hong Kong (No. 9211123). It involved a sample of 507 drug users using purposive sampling. All inmates undergoing drug treatment between July 2017 and March 2018 in all four of the government-run treatment addiction centers in Hong Kong were invited to participate in the study. Prior written consent was obtained from the participants. They anonymously self-administered the questionnaires under the guidance of research assistants inside the centers.

Out of 508 invitations, 507 inmates participated in this study. Approximately three-quarters of them were men (74.8%), while a quarter were women (25.2%). Their ages ranged from 18 to 71 years, of which 29.4% were aged below 30 years. More than half of them (54.4%) used to take drugs more than six times per week. Crystal methamphetamine was the most popular type of drug taken (31.6%). Most of them started taking drugs when they were between 11 and 20 years old (72.0%), and more than half of them took drugs for 10 years or more (55.5%) (Table 1).

### 2.2. Instruments and Analytical Procedures

**Figure 1:**
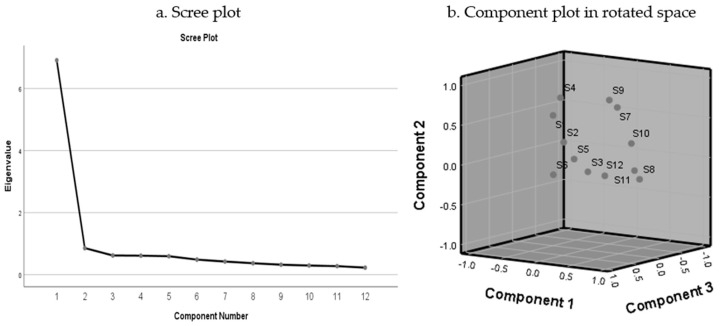
Factor structure of Soulmate Scale depicted in scree plot (**a**) and component plot (**b**). Note: The scree plot shows function of eigenvalues analysis levels in the general neighborhood of the third and fourth component, and the component plot in rotated space visually presents that factor-1 items, S8, S10, S11, and S12, are closely interrelated and located in the lower area at the right side, factor-2 items, S1, S4, S7, and S9, are closely interrelated and located in the upper area at the middle side and factor-3 items, S2, S3, S5, and S6, are closely interrelated and located in the middle of the space.

Based on the literature, we constructed an instrument that includes 12 question items rated with a 5-point measure from 1 (strongly disagree) to 5 (strongly agree). To begin, we calculated the means, standard deviations, and correlation coefficients of the 12 items used to validate the Soulmate Scale (Table 2 and Table 3). We then performed exploratory factor analysis (EFA) of maximum likelihood estimation to figure out the structure of the scale, in which Promax of Kaiser normalization rotation was conducted to allow for the correlation of the respective factors that were extracted [25,26] (Table 4 and Figure 1). This is important, as the items of the Soulmate Scale are expected to be internally coherent and consistent, which should be interrelated and loaded on the latent concept of drug taking as someone’s psychological companion and spiritual solace. In fact, maximum likelihood estimation surpasses principal components analysis because it takes the weights of the variable items on the respective factors to be considered for maximizing the probability of constructing the correlation matrix from a multivariate normally distributed population [27,28].

**Figure 2:**
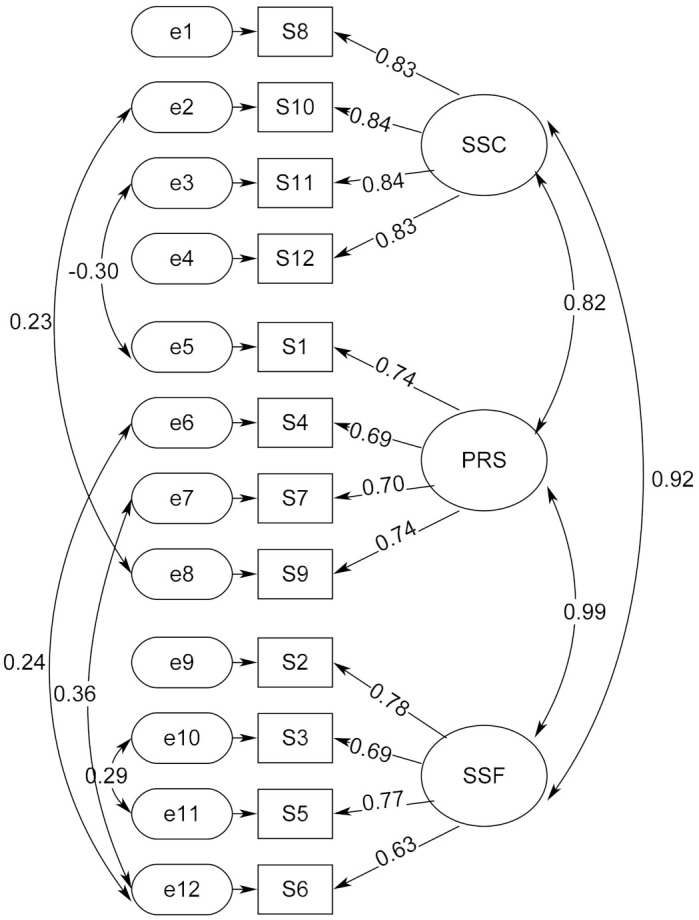
Factor loadings and covariances of the first-order CFA model 1b. Note: SSC denotes the factor of Spiritual Solace and Companionship, PRS means the factor of Psychological Release and Shelter and SSF is the factor of Staunch and Supportive Friendship. The model-data fit is χ^2^/df = 2.451, GFI = 0.934, CFI = 0.965, and RMSEA = 0.077. All factor loadings and covariances were significant at *p* < 0.001, except the covariance between the residuals of S10 and S9 that was significant at *p* < 0.01.

**Figure 3:**
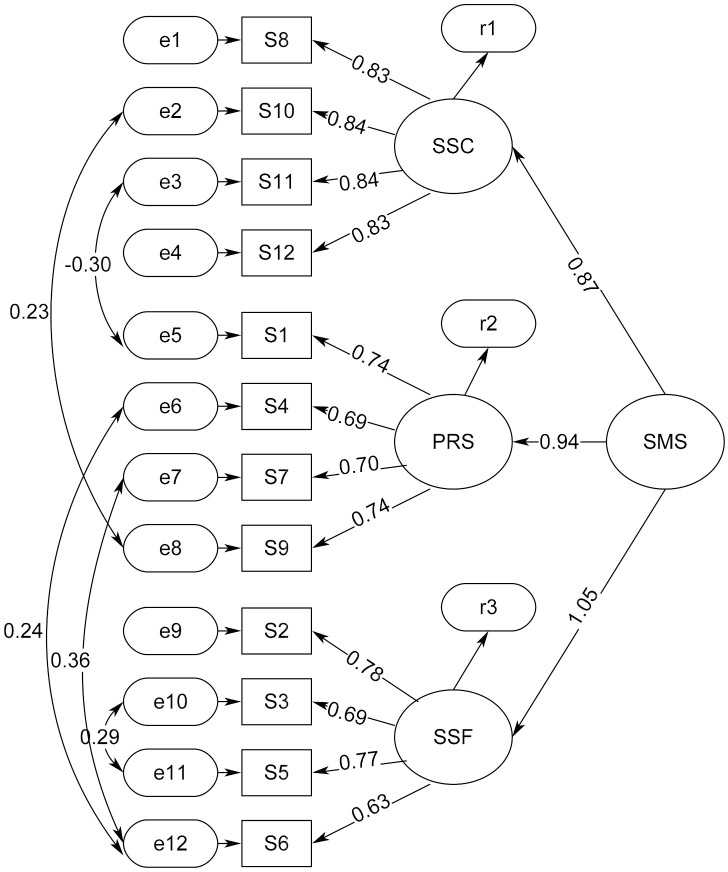
Factor loadings and covariances of the second-order CFA model 1b. Note: SMS means the latent Soulmate Scale, SSC denotes the factor of Spiritual Solace and Companionship, PRS means the factor of Psychological Release and Shelter, and SSF is the factor of Staunch and Supportive Friendship. The model-data fit is χ^2^/df = 2.451, GFI = 0.934, CFI = 0.965, and RMSEA = 0.077. All factor loadings and covariances were significant at *p* < 0.001, except the covariance between the residuals of S10 and S9 that was significant at *p* < 0.01.

The EFA has identified a 3-factor structure, which is later used to perform confirmatory factor analysis (CFA) with sample 2 randomly selected from the total sample. We first performed a first-order CFA structure by loading respective items belonging to the 3 factors generated from EFA (see Figure 2 and Table 5 and Table 6). However, the first-order CFA structure cannot indicate whether the 3 factors are collectively loaded on the common latent construct of the concept of soulmate to expound substances as an agent to provide comfort, support, companionship, reliance, and release to the drug users. Because of this, we performed a second-order CFA to verify whether the 3 factors can significantly and apparently regress on the common latent construct of soulmate (Figure 3 and Table 7).

In order to compare whether the newly established second-order CFA model has external validity across different samples, we imputed and generated 5 datasets by simulation from sample 2 that were used to perform first-order and second-order CFA. The imputation procedure was conducted by Bayesian modelling that is based on the Bayesian interpretation of probability, which assumes parameters are random and that uncertainty regarding parameters can be quantified by probability distributions, hence resampling is attained through the procedure of Markov chain Monte Carlo (MCMC) estimation. Model fit of the second-order CFA model 1b was therefore estimated by the 5 simulated samples to prove its external validity (Table 8).

Lastly, we calculated the Cronbach alpha reliability coefficients of respective factors and the Soulmate Scale (Table 9). In addition, the correlation analysis was used to confirm whether the 3 factors identified are coherently and consistently correlated and whether they come together to have higher correlation coefficients with the Soulmate Scale (Table 10).

## 3. Results

As shown in Table 2, the mean levels of the 12 question items range from 2.258 to 3.242, implying that the participants varied in rating the items between disagree and agree. The standard deviations are all above 1, representing variation of the participants in rating the items. Table 3 shows the Pearson correlation coefficients of the 12 question items, in which the levels of correlation coefficients ranged from *r* = 0.353 and *r* = 0.691, *p* < 0.01, with the former being the correlation of E10 and E17 and the latter being the correlation of E19 and E20. In fact, the range of correlation coefficients are universally standardized in-between −1 and 1. The magnitude of correlation coefficients in the present study was found all significantly ≥0.350 and ≤0.800, with *p* < 0.01, and the item-total correlation coefficient of the 12 measurement items was *r*_tot_ = 0.540, with T^2^ (df1, df2) = 420.654 (11, 436), *p* < 0.001, connoting their highly adequate and strong intercorrelations. Moreover, this range of correlation coefficients does not constitute a problem of multicollinearity and common-method variance, which is supported by the moderate and discernible level of the average correlation coefficient, *r*_average_ = 0.539, and the equation for the average correlation coefficient is

(1) $$\bar{r}=\frac{\sum (n_{i}-1)r_{i}}{\sum n_{i}-k}$$

where *k* is the number of individual *r* coefficients being aggregated and *n_i_* is the ith sample *r* that is modeled to compute the average level of standardized correlations.

For validating the Soulmate Scale, EFA of maximum likelihood estimation was performed. The EFA showed a 3-factor model solution of the scale. Table 4 shows its structure and factor loadings. Four items are manifestly loaded on factor 1 that includes S8, S10, S11, and S12; four items are loaded on factor 2 that includes S1, S4, S7, and S9; and the remaining four items are apparently loaded on factor 3 that includes S2, S3, S5, and S6. This 3-factor model solution explained 69.921% of the total variance. Both Kaiser–Meyer–Olkin value, KMO = 0.940, and Bartlett’s test of sphericity, χ^2^ (df) = 1873.624(66), *p* < 0.001, suggest the factoring procedure to be of sampling and correlation adequacy. When we inspected the contents of the respective items loaded on their different factors, three main factorial themes emerged. Specifically, the items loaded on factor 1 describe substances as an agent to make the drug users feel solaced and companied in their spiritual and inner world; hence, factor 1 is here termed Spiritual Solace and Companionship (SSC). Items belonging to factor 2 mention that the users’ emotional distress can be released and that substances can be used as a shelter to let them psychologically rest; hence, factor 2 is defined as Psychological Release and Shelter (PRS). Lastly, items of factor 3 describe substances as a friend to give support, courage, and acceptance to the users; therefore, it is named Staunch and Supportive Friendship (SSF).

Convincingly, the strengths of the factor loadings are all well beyond the stringent required level of λ ≥ 0.40, in which factor loadings of SSC ranged from 0.567 to 0.863, factor loadings of PRS ranged from 0.619 to 0.805, and factor loadings of SSF were between 0.512 and 0.810. In fact, Figure 1 portrays that scree plot supports this 3-factor solution structure, in which the function of eigenvalues analysis levels out in the region of the third and fourth component (Figure 1a), and component plot in rotated space also apparently presents that different items loaded on their respective factors are closely interrelated and are at different locations of the common space (Figure 1b). Taken together, this 3-factor structure was used to perform confirmatory factor analysis (CFA) with sample 2.

For CFA, we first performed a first-order CFA structure by loading respective items belonging to the 3 factors generated from EFA. Table 5 shows that this initial CFA model of no covariance did not fit the data adequately (model 1); although the χ^2^/df ratio and CFI were at acceptable levels, χ^2^/df = 3.839 and CFI = 0.924, the levels of GFI and RMSEA were inadequate, GFI = 0.886, and RMSEA = 0.107. Checking the modification indexes of the model, the residuals of item S11 and S1, S7 and S12, and S10 and S11 were required to covary. After setting three covariances between these residuals, the first-order CFA model became a much better fit to the data and reached an acceptable level (model 1a), in which χ^2^/df = 2.837, GFI = 0.922, CFI = 0.954, RMSEA = 0.086. Nevertheless, modification indexes were further required to set residuals of item S9 and S10, as well as S4 and S6 to covary. By setting these residuals as covariances, the first-order CFA attained a well adequate level of model-data fit (model 1b), in which χ^2^/df = 2.451, GFI = 0.934, CFI = 0.965, RMSEA = 0.077. In fact, the much lower levels of AIC and BCC further support that model 1b is preferable to the other two CFA models.

Table 6 and Figure 2 present the factor loadings of the first-order CFA model 1b, in which all regression coefficients are beyond the required threshold of λ ≥ 0.40. For the factor of SSC, the factor loadings ranged from 0.830 to 0.843, the factor loadings of PRS ranged from 0.691 to 0.739 and the factor loadings of SSF had a range from 0.630 to 0.784.

Although the first-order CFA model 1b shows a good fit to the data and obtained high levels of factor loadings, it cannot indicate whether the three factors of SSC, PRS, and SSF are collectively loaded on the common latent construct of the concept of soulmate. Therefore, a second-order CFA was performed to verify whether the three factors can significantly and apparently regress on the common latent construct of soulmate. Figure 3 presents the second-order CFA model 1b, in which all the first-order factor loadings and covariances were maintained intact. Specifically, we can see that the second-order loadings from SSC, PRS, and SSF to the soulmate latent construct SMS were strong and significant. Table 6 and Table 7 present the first-order and the second-order CFA model 1b in which the second-order loading from SMS to SSC is 0.872, from SMS to PRS is 0.938, and from SMS to SSF is 1.054, while all the first-order factor loadings remained unchanged. Model fit of the second-order CFA model 1b was estimated by the five simulated samples aforementioned to prove its external validity. Table 8 presents that the second-order CFA model 1b attained acceptable model-data fit across the five samples, in which all the models had the χ^2^/df ratio ≤ 3, GFI and CFI ≥ 0.90 and RMSEA ≤ 0.08, with the best fit model generated from sample 4, in which the model fit indexes were χ^2^/df ratio = 2.406, GFI = 0.935, CFI = 0.966, RMSEA = 0.075, AIC = 174.693, and BCC = 178.248.

Table 9 reports the Cronbach alpha reliability coefficients of respective factors and the total Soulmate Scale, in which all are above the required level of α ≥ 0.70, with a range from 0.816 to 0.933. In fact, Hotelling’s *T^2^* multivariate tests supported the question items that are aggregated to construct the respective factors and the total Soulmate Scale have different multivariate means and variances. Moreover, the correlation analysis was performed to confirm whether the respective factors of Spiritual Solace and Companionship, Psychological Release and Shelter, and Staunch and Supportive Friendship are coherently and consistently correlated together, which come together to have higher correlation coefficients with the Soulmate Scale. Hence, it is expected that the levels of correlation coefficients of the respective factors should range between *r* ≥ 0.300 and *r* ≤ 0.800, and the levels of correlation coefficients of the respective factors in relation to the Soulmate Scale should be *r*s ≥ 0.800. Table 10 shows that the correlation coefficients of the respective factors ranged from *r* = 0.697 to *r* = 0.791, *p*s < 0.001 and the correlation coefficients of the respective factors with the Soulmate Scale ranged from *r*s = 0.901 and 0.936, *p*s < 0.001. Conclusively, the factors of Spiritual Solace and Companionship, Psychological Release and Shelter, and Staunch and Supportive Friendship and the Soulmate Scale are empirically confirmed to have reliability of internal consistency and consistency.

## 4. Discussion

Substance users use substances to satisfy different psychological factors, such as to seek and obtain psychological release and shelter, staunch and supportive friendships, and spiritual solace and companionship. Such experience resembles seeking a soulmate for receiving comfort, a sense of security and satisfaction to relieve feelings of loneliness [22,24]. Substance users perceive drugs as spiritually connected partners who can fulfil their needs for intimacy and are challenging to cut off [22,24]. This supports recent research that drug users’ psychological experience constitutes a significant factor of their persistent engagement in drug-taking [13]. The need for belongingness, or relatedness, is a basic need for human beings [29,30,31]. People are inclined to build interpersonal relationships until they reach a certain quantity and quality of relatedness (relationships with mutual affection, intimacy, and stability); if deprived, they will display stress, maladjustment, and mental health issues [29]. Consistent with this position, recent research has found that having adverse relationships with significant others (e.g., family and spouses) that lack love, care, and support caused people to take drugs as a way to compensate for the lack of connectedness, and that the loss of companions was a significant factor in drug relapse to relieve despair and emptiness [8,13]. This reflects that the substance users’ dependence on drugs is a manifestation of their need for warmth, comfort, and affiliation, which can be attained through having a “soulmate” experience.

As revealed by the five stages of hidden drug abuse in a recent research [8], the initial stage of substance use is a means of obtaining relatedness with peers through social events. To drive away the boredom and low mood in everyday life, individuals look for social playmates and companionship. Although the sense of brotherhood and sisterhood among drug peers can give them warmth and a feeling of acceptance at the earlier stage, the more regularly they take drugs, the more alienated and lonelier they become. Eventually, to escape from any conflict, suspicion, or possible discrimination, they withdraw from and disconnect with any social network. As a result, substance use becomes their connection to a soulmate, who is a spiritually trusted partner. This is the only way of obtaining comfort to satisfy their needs for connectedness and release their strong sense of loneliness. Loneliness is not only being alone but a subjective experience of psychological and social isolation comprising a negative state, such as depression or anxiety, which is emotional loneliness, and a perception of deficiency in social networks or relationships, which is social loneliness [32,33,34]. Therefore, the validation of this soulmate scale for substance addiction and loneliness is necessary to assess the experience of substance users’ social and emotional loneliness and their spiritual consolation to drugs. Factor 1 of the scale, Spiritual Solace and Companionship (SSC), is the measure of their spiritual attachment to drugs. Factor 2, Psychological Release and Shelter (PRS), is their experience of emotional loneliness, and Factor 3, Staunch and Supportive Friendship (SSF), is their experience of social loneliness. The Mental Health Foundation in England and Scotland published a report entitled “The Lonely Society?” in 2010, which revealed that loneliness has become even more prevalent in contemporary societies [35]. The increase in loneliness in a society may further worsen the situation of substance addiction.

Empirical research and validation of relevant measures to assess substance addiction and loneliness are definitely helpful for early intervention and prevention. The present study develops a Soulmate Scale to measure substance addiction and loneliness. Results show that this three-factor scale is a valid and reliable measure of the psychological sustenance and consolation of drugs to substance users. The construct of “soulmate” performed by drugs from the perspective of substance users is justified. One limitation is that this study is performed based on a Hong Kong drug user sample. To confirm the fitness of the Soulmate Scale to measure substance addiction and loneliness, further research is required to examine the validity and reliability of the Soulmate Scale when adapted to countries of different cultures and for other substance user samples.

## 5. Conclusions

To date, research exploring the “soulmate” experience of drug-taking among substance users is still scarce. The present study offers additional support for understanding the drug-taking experience of substance users from their perspective. Losing respect, recognition, and acceptance from significant others during the process of recovery imposes further stress on drug users to rely on drugs as soulmates [36,37]. The validity and reliability of the Soulmate Scale make it a useful tool to assess the underlying factors that affect substance users to persistently take drugs. Assessing their inner-sense and their perception of drugs will provide valuable insights for formulating corresponding intervention plans to achieve drug abstinence, such as choosing suitable therapy in individual counselling to deal with substance users’ emotional loneliness, and group intervention to address their social loneliness by helping them to rebuild constructive social networks to facilitate their social reintegration and detach from the drug users network [38]. It is hoped that the scale can be applied in social service settings to assess the substance users’ psychological dependence on drugs. Future research can be performed to investigate the significance of such assessment in treatment outcomes.

## Figures and Tables

**Table 1:** Participants’ demographic and drug use data.

Variable	*n*	%
Gender (*n* = 507)		
Male	379	74.8
Female	128	25.2
Age (*n* = 500)		
18–20	19	3.8
21–30	128	25.6
31–40	149	29.8
41–50	168	33.6
51–71	36	7.2
Frequency of taking drugs (*n* = 500)		
Less than once per month	19	3.8
Once per month	18	3.6
2–3 times per month	59	11.8
1–2 times per week	55	11.0
3–6 times per week	77	15.4
More than 6 times per week	272	54.4
Types of drugs taken (*n* = 507)		
Crystal methamphetamine	333	65.7
Cocaine	159	31.4
Ketamine	121	23.9
Heroin	143	28.2
Nimetazepam	54	10.7
Cannabis	80	15.8
Ecstasy	39	7.7
Others (triazolam, methaqualone, cough medicine)	124	37.2
Age at first drug taking (*n* = 482)		
10 years old or below	3	0.6
11–20 years old	347	72.0
21–30 years old	92	19.1
31–50 years old	39	8.1
51 years old or above	1	0.2
Duration of taking drugs (*n* = 476)		
Fewer than 3 years	45	8.1
Between 3 and 4.99 years	61	11.0
Between 5 and 9.99 years	141	25.4
Between 10 and 20 years	221	39.8
More than 20 years	87	15.7

**Table 2:** Descriptive statistics of the question items used for validation of the Soulmate Scale.

Item	Contents	Mean	SD	Range
S1	Drugs can let me release my suppressed emotions	3.153	1.065	1–5
S2	Drugs are a friend who can accompany me at any time to solve loneliness	2.812	1.123	1–5
S3	Drugs are a friend who can make me change myself successfully	2.619	1.134	1–5
S4	Drugs are a friend I love and hate	3.242	1.173	1–5
S5	Drugs are a friend who can give me courage	2.577	1.090	1–5
S6	Drugs are a friend who will not refuse or criticize me	2.980	1.140	1–5
S7	Drugs are my refuge, protecting me from being hurt by negative emotions or events	2.814	1.111	1–5
S8	Drugs are an angel	2.258	1.151	1–5
S9	When I am hurt or sad, drugs can fill the wounds of my heart, reducing my pain	2.966	1.133	1–5
S10	Drugs give me the feeling of being understood and cared for	2.558	1.093	1–5
S11	There is nothing in my world, drugs are my only soul partner	2.360	1.160	1–5
S12	Drugs are a friend who will not leave me	2.540	1.202	1–5

Note. For avoiding acquaintance and common method variance, the question items are dispersed in different parts of the questionnaire without sequencing. The question items are rated with a 5-point measure, in which 1 = strongly disagree, 2 = disagree, 3 = neutral, 4 = agree, and 5 = strongly agree.

**Table 3:** Pearson correlation of the question items adopted to validate the Soulmate Scale.

Item	S1	S2	S3	S4	S5	S6	S7	S8	S9	S10	S11	S12
S1	--											
S2	0.645 **	--										
S3	0.488 **	0.527 **	--									
S4	0.506 **	0.511 **	0.467 **	--								
S5	0.527 **	0.606 **	0.649 **	0.517 **	--							
S6	0.453 **	0.504 **	0.477 **	0.478 **	0.532 **	--						
S7	0.497 **	0.536 **	0.474 **	0.492 **	0.558 **	0.584 **	--					
S8	0.425 **	0.529 **	0.544 **	0.353 **	0.589 **	0.448 **	0.462 **	--				
S9	0.584 **	0.566 **	0.482 **	0.542 **	0.530 **	0.502 **	0.587 **	0.532 **	--			
S10	0.537 **	0.573 **	0.589 **	0.464 **	0.6 **	0.503 **	0.559 **	0.670 **	0.661 **	--		
S11	0.392 **	0.552 **	0.587 **	0.362 **	0.572 **	0.489 **	0.566 **	0.628 **	0.528 **	0.691 **	--	
S12	0.444 **	0.604 **	0.516 **	0.422 **	0.581 **	0.531 **	0.510 **	0.641 **	0.573 **	0.654 **	0.687 **	--

** *p* < 0.01.

**Table 4:** Factor loadings of exploratory factor analysis (EFA) by Promax of Kaiser normalization rotation method.

Item	Contents	Factor 1	Factor 2	Factor 3
S1	Drugs can let me release my suppressed emotions		0.619	0.426
S2	Drugs are a friend who can accompany me at any time to solve loneliness		0.324	0.547
S3	Drugs are a friend who can make me change myself successfully	0.389		0.512
S4	Drugs are a friend I love and hate		0.805	
S5	Drugs are a friend who can give me courage			0.569
S6	Drugs are a friend who will not refuse or criticize me			0.810
S7	Drugs are my refuge, protecting me from being hurt by negative emotions or events	0.357	0.689	
S8	Drugs are an angel	0.863		
S9	When I am hurt or sad, drugs can fill the wounds of my heart, reducing my pain		0.779	
S10	Drugs give me the feeling of being understood and cared for	0.679		
S11	There is nothing in my world, drugs are my only soul partner	0.805		
S12	Drugs are a friend who will not leave me	0.567		

Note. Factor 1 is termed Spiritual Solace and Companionship (SSC), which includes item S8, S10, S11, and S12; factor 2 is defined as Psychological Release and Shelter (PRS), which includes item S1, S4, S7, and S9; and factor 3 is named Staunch and Supportive Friendship (SSF), which includes items S2, S3, S5, and S6.

**Table 5:** Model fit of the first-order confirmatory factor analysis (CFA) models of the Soulmate Scale.

Model	χ^2^	df	*P*-Value	χ^2^/df	GFI	CFI	RMSEA	AIC	BCC
CFA of no covariance (Model 1)	195.809	51	<0.001	2.839	0.886	0.924	0.107	249.809	252.809
CFA of 3 residual covariances (Model 1a)	136.194	48	<0.001	2.837	0.922	0.954	0.086	196.194	199.528
CFA of 5 residual covariances (Model 1b)	112.738	46	<0.001	2.451	0.934	0.965	0.077	176.738	180.294

Note. For model fit indexes, χ^2^ = identified model chi-square value, df = degree of freedom, χ^2^/df = chi-square value to degree of degree ratio, GFI = Goodness-of-Fit Index, CFI = Comparative Fit Index, RMSEA = Root Mean Square Error of Approximation, AIC = Akaike’s Information Criterion, and BCC = Bayes Information Criterion.

**Table 6:** Factor loadings of the first-order CFA model 1b.

Path	Coefficient	Standard Error	Critical Ratio
SSC-> S12	0.830	--	--
SSC-> S11	0.841	0.061	15.84 ***
SSC-> S10	0.843	0.057	15.917 ***
SSC-> S8	0.830	0.066	15.529 ***
PRS-> S9	0.736	--	--
PRS-> S7	0.704	0.09	10.687 ***
PRS-> S4	0.691	0.094	10.482 ***
PRS-> S1	0.739	0.082	11.204 ***
SSF-> S6	0.630	--	--
SSF-> S5	0.769	0.119	10.134 ***
SSF-> S3	0.687	0.124	9.284 ***
SSF-> S2	0.784	0.124	10.285 ***

Note: SSC denotes the factor of Spiritual Solace and Companionship, PRS means the factor of Psychological Release and Shelter and SSF is the factor of Staunch and Supportive Friendship. The regression weights of SSC-> S12, PRS-> S9, and SSF-> S6 were set to 1, hence standard error and critical ratio were not estimated. The model fit is χ^2^/df = 2.451, GFI = 0.934, CFI = 0.965, and RMSEA = 0.077. *** *p* < 0.001.

**Table 7:** Factor loadings of the second-order CFA model 1b.

Path	Coefficient	Standard Error	Critical Ratio
SMS-> SSC	0.872	0.066	13.164 ***
SMS-> PRS	0.938	0.065	12.131 ***
SMS-> SSF	1.054	0.068	10.957 ***
SSC-> S12	0.830	--	--
SSC-> S11	0.841	0.061	15.84 ***
SSC-> S10	0.843	0.057	15.917 ***
SSC-> S8	0.830	0.066	15.529 ***
PRS-> S9	0.736	--	--
PRS-> S7	0.704	0.09	10.687 ***
PRS-> S4	0.691	0.094	10.482 ***
PRS-> S1	0.739	0.082	11.204 ***
SSF-> S6	0.630	--	--
SSF-> S5	0.769	0.119	10.134 ***
SSF-> S3	0.687	0.124	9.284 ***
SSF-> S2	0.784	0.124	10.285 ***

Note. SMS means the latent Soulmate Scale, SSC denotes the factor of Spiritual Solace and Companionship, PRS means the factor of Psychological Release and Shelter, and SSF is the factor of Staunch and Supportive Friendship. The regression weights of SSC-> S12, PRS-> S9 and SSF-> S6 were set to 1, hence standard error and critical ratio were not estimated. The model fit is χ^2^/df = 2.451, GFI = 0.934, CFI = 0.965, and RMSEA = 0.077. *** *p* < 0.001.

**Table 8:** Model fit of the second-order CFA model 1b from simulated samples.

Model Sample	χ^2^	df	*p*-Value	χ^2^/df	GFI	CFI	RMSEA	AIC	BCC
Imputed Sample 1	112.304	46	<0.001	2.441	0.933	0.965	0.076	176.304	179.860
Imputed Sample 2	117.743	46	<0.001	2.560	0.931	0.963	0.079	181.743	185.299
Imputed Sample 3	113.544	46	<0.001	2.468	0.933	0.965	0.077	177.544	181.099
Imputed Sample 4	110.693	46	<0.001	2.406	0.935	0.966	0.075	174.693	178.248
Imputed Sample 5	116.401	46	<0.001	2.532	0.931	0.963	0.079	180.401	184.017

Note. For model fit indexes, χ^2^ = identified model chi-square value, df = degree of freedom, χ^2^/df = chi-square value to degree of degree ratio, GFI = Goodness-of-Fit Index, CFI = Comparative Fit Index, RMSEA = Root Mean Square Error of Approximation, AIC = Akaike’s Information Criterion, and BCC = Bayes Information Criterion.

**Table 9:** Reliability coefficients of the factor of Spiritual Solace and Companionship, Psychological Release and Shelter, and Staunch and Supportive Friendship and the Soulmate Scale.

	Sample 1		Sample 2		Total Sample	
	α	*T* ^2^	α	*T* ^2^	α	*T* ^2^
SSC	0.867	17.616 ***	0.905	9.953 ***	0.887	26.129 ***
PRS	0.828	20.129 ***	0.816	11.283 ***	0.821	28.415 ***
SSF	0.837	8.784 ***	0.823	19.828 ***	0.830	27.067 ***
SMS	0.933	24.908 ***	0.933	17.184 ***	0.933	41.004 ***

Note. SSC = Spiritual Solace and Companionship, PRS = Psychological Release and Shelter, SSF = Staunch and Supportive Friendship; SMS = Soulmate Scale. *T^2^ =* Hotelling’s**T*^2^* multivariate tests, which are used to reject the *H*_0_ hypothesis that all items on the respective factors and the total Soulmate Scale have the same mean and variance levels. *** *p* < 0.001.

**Table 10:** Correlation coefficients of the factors of Spiritual Solace and Companionship, Psychological Release and Shelter, and Staunch and Supportive Friendship and the Soulmate Scale in the total sample.

	SSC	PRS	SSF	SMS
SSC	--			
PRS	0.697 ***	--		
SSF	0.785 ***	0.791 ***	--	
SMS	0.909 ***	0.901 ***	0.936 ***	--

Note. SSC = Spiritual Solace and Companionship, PRS = Psychological Release and Shelter, SSF = Staunch and Supportive Friendship. SMS = Soulmate Scale. *** *p* < 0.001.
